# CD36-Mediated Lipid Metabolic Reprogramming in Hepatocellular Carcinoma: Crosstalk With Immunity, Ferroptosis, and Therapeutic Resistance

**DOI:** 10.7759/cureus.116886

**Published:** 2026-09-25

**Authors:** Ankit Kumar Bijaraniya, Tao Lide

**Affiliations:** 1 Department of General Surgery, Yangzhou University, Yangzhou, CHN; 2 Department of General Surgery, Affiliated Hospital of Yangzhou University, Yangzhou University, Yangzhou, CHN

**Keywords:** cd36, ferroptosis, hepatocellular carcinoma, lipid metabolism reprogramming, metabolic-immune checkpoint, therapeutic resistance, tumor immunometabolism

## Abstract

Hepatocellular carcinoma (HCC) is a leading cause of cancer death and is associated with metabolic dysfunction and chronic liver disease. Lipid metabolic reprogramming is a key hallmark of HCC, and CD36, a fatty acid translocase and scavenger receptor, has emerged as a central regulator of this process. This review investigated the multifaceted roles of CD36 in HCC pathogenesis. CD36 facilitates long-chain fatty acid uptake and affects oncogenic signaling and the tumor immune microenvironment. CD36-mediated lipid accumulation promotes cytotoxic T-cell function and macrophage M2 polarization and drives immune evasion. Likewise, CD36 modulates ferroptosis sensitivity by influencing polyunsaturated fatty acid-induced lipid peroxidation and redox balance. Through metabolic plasticity and oxidative stress adaptation, CD36 also contributes to resistance to chemotherapy, targeted therapies, and immunotherapy. In conclusion, CD36 serves as a critical multifunctional regulator linking lipid metabolism, ferroptosis, immunomodulation, and therapy resistance in HCC, positioning it as a promising therapeutic target for improving clinical outcomes. However, clinical translation remains challenging due to the broad tissue expression of CD36 and the lack of large-scale clinical validation. Hence, future research should focus on clinical validation of CD36, mechanistic elucidation, selective inhibitor development, combination therapy evaluation, and multi-omics-guided personalized HCC treatment.

## Introduction and background

Hepatocellular carcinoma (HCC) is the most common type of primary liver cancer and a leading cause of cancer-related deaths worldwide [1]. Although the survival of patients with HCC has improved significantly as a result of surgical resection, liver transplantation, molecular targeting, local interventions, and immunotherapy, the overall survival of patients with HCC remains unsatisfactory because of many challenges, including delayed diagnosis, high recurrence rates, high metastatic potential, tumor heterogeneity, and resistance to available therapies [2]. Metabolic reprogramming is a fundamental feature of hepatocarcinogenesis, enabling transformed hepatocytes to meet the increased demands for energy, biosynthetic precursors, and redox balance during tumor initiation and progression. In particular, dysregulated lipid metabolism can assist cancer cell proliferation and survival by altering fatty acid uptake, synthesis, storage, and utilization while influencing oncogenic signaling and interactions with the tumor microenvironment. Although the incidence of chronic hepatitis B virus (HBV) and hepatitis C virus (HCV) infection has decreased in different regions, these infections remain important risk factors for HCC development and contribute significantly to the global burden of HCC, alongside alcoholic liver disease, metabolic dysfunction-associated steatotic liver disease (MASLD), and its inflammatory subtype, metabolic dysfunction-associated steatohepatitis (MASH) [3].

MASLD represents a chronic metabolic disease state that generates a permissive inflammatory and fibrotic environment for hepatocarcinogenesis and metabolic reprogramming in HCC. Interestingly, the link between metabolic dysregulation and HCC development has been growing in strength, and a number of research studies on metabolic reprogramming as a fundamental driver of HCC progression have emerged. Metabolic reprogramming is a hallmark of cancer that enables the adaptation of tumor cells to a challenging microenvironment that includes hypoxia, oxidative stress, nutrient deprivation, and immune surveillance [4]. These adaptive metabolic alterations in HCC are remarkably similar to those investigated in other cancer types and serve primarily to assist tumor progression and survival under microenvironmental stress rather than acting as a direct driver of the initial carcinogenic event. Previous studies on cancer metabolism were primarily focused on glucose metabolism and the Warburg effect, which established the classic view that cancer cells prefer aerobic glycolysis for rapid energy production and biomass synthesis. However, this focus has progressively expanded, and recent studies highlight the important role of lipid metabolism in the process of tumorigenesis and progression [5]. This phenomenon is especially relevant in the context of HCC, as the liver is the major organ responsible for lipid synthesis, storage, transport, and catabolism. HCCs exhibit marked differences in fatty acid uptake, de novo lipogenesis, β-oxidation, lipid storage, and cholesterol metabolism compared to normal hepatocytes, which contribute to their rapid growth, membrane biosynthesis, metastasis, and metabolic stress adaptation [6]. In addition, lipid accumulation and metabolic plasticity confer tolerance to oxidative stress, angiogenesis, immune evasion, and resistance to anticancer therapies, ultimately leading to tumor invasion and poor clinical prognosis [7].

Among the key regulators involved in lipid metabolic adaptation, cluster of differentiation 36 (CD36), also known as fatty acid translocase, has become an important mediator of tumor-associated lipid metabolism [8]. CD36 is a multifunctional scavenger receptor expressed in a variety of cells, including hepatocytes, adipocytes, endothelial cells, macrophages, platelets, and immune cells, and plays a role in long-chain fatty acid uptake, lipid transport, inflammatory signaling, cell adhesion, and metabolic homeostasis [9]. There is growing evidence that the expression of CD36 has a close association with the prognosis of different types of cancers, including breast, gastric, colorectal, ovarian, pancreatic, and HCC (Table 1) [10]. CD36 expression is linked to the accumulation of lipids, metastatic capability, epithelial-mesenchymal transition (EMT), maintenance of stem cell properties, and poor prognosis in patients with HCC [11].

Recent investigations have shown that CD36-mediated fatty acid uptake can activate several oncogenic signaling pathways, including the PI3K/AKT/mTOR, MAPK, STAT3, TGF-β, and Wnt/β-catenin pathways, thereby promoting tumor cell proliferation, invasion, angiogenesis, and metastatic resistance (Table 2) [12]. Moreover, CD36 bestows metabolic flexibility on tumor cells, enabling them to use exogenous fatty acids as a metabolic source in the absence of nutrients. This metabolic switch is beneficial to the survival of tumors under hypoxia, oxidative stress, and anticancer treatment [13]. Previous studies demonstrated that overexpression of CD36 is linked to the maintenance and metastatic colonization of cancer stem cells, thus reinforcing its role in cancer recurrence and progression. The biological function of CD36 in HCC is complex and controversial, given its oncogenic functions. Some investigations observed that CD36 may have different effects depending on the metabolic state or tumor microenvironment, tumor stage, immune composition, and nutrient supply. These inconsistencies highlight the need to better understand CD36's role in HCC.

In addition to its role in lipid transport, CD36 has recently been identified as a key player in tumor immunometabolism and microenvironment remodeling [8]. The HCC microenvironment is very complex and includes a large number of metabolic interactions between tumor cells, immune cells, stromal cells, and endothelial cells. CD36-mediated over-uptake of lipids can cause lipid accumulation in immune cells, resulting in promoted cytotoxic T-cell function, impaired T-cell exhaustion, and macrophage polarization [11]. In addition, lipid metabolism via CD36 is linked to inflammatory signaling, immune evasion, and immune checkpoint blockade resistance. These results have led to the novel idea of CD36 as a new metabolic-immune checkpoint connecting tumor metabolism, immune regulation, and therapeutic response.

Another rapidly developing area of research is the relationship between CD36, oxidative stress, and ferroptosis. Ferroptosis is a novel type of iron-dependent cell death, defined by an increase in lipid peroxidation and accumulation of reactive oxygen species (ROS) [14]. Recent data show that digestion can be modulated by CD36-dependent polyunsaturated fatty acids through lipid peroxidation, redox homeostasis, and antioxidant defense pathways [11]. Although induction of fermentation has been proposed as a therapeutic approach for HCC, the mechanism of interaction between CD36, lipid peroxidation, fermentation vulnerability, and treatment resistance remains to be fully understood. Moreover, lipid accumulation has been reported to have both pro-survival and pro-ferroptosis effects, further complicating the metabolic signaling of CD36 in HCC [10].

Notably, the development of different technologies and treatments has increased interest in research targeting CD36 in cancer. The combination of lipidomics, metabolomics, single-cell RNA sequencing, spatial transcriptomics, and multi-omics has greatly enhanced the understanding of metabolic heterogeneity and the dynamics of the tumor microenvironment in HCC. Recent preclinical studies demonstrated that pharmacological or genetic ablation of CD36 could suppress fatty acid uptake, inhibit metastasis, sensitize cells to ferroptosis, enhance antitumor immunity, and boost the efficacy of therapy [11]. Moreover, therapies that combine the use of CD36 inhibitors with targeted therapies, processed inhibitors, and immune checkpoint inhibitors have demonstrated promising antitumor activity in experimental models. However, there are a number of key restrictions that limit clinical translation. At present, most of the evidence is based on in vitro studies and animal models, and large-scale validation in patients has not yet been conducted. Moreover, CD36 is widely distributed in tissues, so systemic inhibition of CD36 may impair normal lipid metabolism and immune function [15]. Furthermore, developing stable, highly selective monoclonal antibodies against CD36 that effectively target the tumor microenvironment without disrupting systemic lipid homeostasis in healthy tissues remains a key clinical objective. In addition, the efficacy of long-term treatment with CD36-targeted drugs might be affected by compensatory metabolic pathways and metabolic redundancy.

The topic of cancer metabolism, particularly lipid metabolic reprogramming in HCC, has been explored in various investigations. However, most previous studies have primarily emphasized general metabolic changes or described CD36 primarily as a fatty acid transporter. However, comprehensive reviews on the mechanisms of CD36-derived drugs integrated with immunometabolism, processing, oxidative stress adaptation, remodeling of the tumor microenvironment, metabolic plasticity, and resistance to HCC treatment remain limited. In addition, there is no systematic summary of the translational applications of recent advances in single-cell sequencing, spatial metabolomics, and precision oncology in the context of CD36-targeted therapy. Thus, an extensive mechanistic review that brings together these emerging fields of study is urgently required.

Therefore, the present review highlights the current knowledge of CD36-dependent lipid metabolic reprogramming in HCC and its implications for tumor progression, metabolic plasticity, immunometabolism, ferroptosis, oxidative stress adaptation, and treatment resistance. Furthermore, the article examines classes of drugs directed against CD36, such as pharmacological inhibitors, antibody drugs, nanomedicines, and combination drugs. The review incorporates molecular mechanisms, interactions with the tumor microenvironment, multi-omics approaches, and insights from the field of translational medicine to offer a comprehensive overview of the multifaceted functions of CD36 in HCC and set the stage for future research in the realm of targeted liver cancer therapy.

## Review

Review methodology

Literature Search Strategy

To collect and evaluate published research on CD36-mediated lipid metabolic reprogramming in HCC, we systematically searched multiple scientific databases, including PubMed/MEDLINE, Web of Science, Scopus, Embase, and Google Scholar, for relevant articles up to the most recent publication date. We also carried out an active search for other relevant studies by scanning the reference lists of articles included in the analysis and recently published articles.

The search strategy included Medical Subject Headings (MeSH) and free-text keywords associated with CD36, HCC, lipid metabolism, ferroptosis, tumor immunity, oxidative stress, and drug resistance. The search terms included CD36, fatty acid translocation, HCC, lipid remodeling, ferroptosis, tumor microenvironment, immunometabolism, oxidative stress, and drug resistance. Boolean operators “AND” and “OR” were used to enhance the sensitivity and specificity of the search.

Study Selection and Eligibility Criteria

Relevant studies were screened according to predefined inclusion criteria. Original research articles investigating the biological, molecular, metabolic, immunological, and therapeutic roles of CD36 in HCC were eligible for inclusion. Eligible studies included experimental and clinical studies, including in vitro studies, animal experiments, translational analyses, and patient studies, to provide a comprehensive overview of the current understanding of the mechanisms of CD36-related HCC.

Studies reporting a direct correlation with CD36-related pathways in HCC or with insufficient mechanistic or translational information were excluded.

Data Collection and Thematic Organization

Relevant information was extracted from studies meeting the inclusion criteria and organized according to the main themes of this review. This review aims to describe studies that have examined the role of CD36 in fatty acid uptake, metabolic plasticity, lipid accumulation, processing, oxidative stress adaptation, tumor microenvironment remodeling, immune escape, and drug resistance in HCC.

The evidence collected is presented in several thematic sections: structure and physiological role of CD36, reprogramming of lipid metabolism, oncogenic signaling pathways, ferroptosis and oxidative stress, immunometabolic regulation, treatment resistance, and new precision medicine strategies. In addition, the latest advances in lipidomics, metabolomics, single-cell RNA sequencing, spatial transcriptomics, and multi-omics are reviewed to highlight developments in CD36-related HCC research.

All included studies were carefully evaluated using criteria for scientific quality, experimental design, mechanistic insight, translational relevance, and clinical relevance. We emphasized the best mechanistic studies, clinically validated evidence, and the most recent multi-omics studies. Because CD36 is a context-dependent molecule that plays a contradictory role in tumor progression, processing sensitivity, and immune regulation, we performed a comparative analysis of conflicting results to present a balanced interpretation of the available evidence.

Scope and Purpose of the Review

This review aims to integrate current knowledge about the multifaceted roles of CD36 in HCC, particularly its role in lipid metabolism adaptation, tumor progression, ferroptosis, immune remodeling, and treatment resistance. Furthermore, this review explores emerging CD36-targeted therapy strategies and looks ahead to future research directions in metabolism-based precision oncology for HCC treatment.

Results

Overview of the Included Literature

The literature suggests that CD36 plays multiple roles in HCC progression, participating in processes such as lipid metabolic reprogramming, tumor microenvironment remodeling, ferroptosis regulation, oxidative stress adaptation, and treatment resistance. Included studies comprise in vitro experiments, animal experiments, translational analyses, bioinformatics studies, and clinical observations evaluating CD36 expression and function in HCC.

Most studies consistently report abnormally upregulated CD36 expression in HCC tissues compared to adjacent normal liver tissues [16]. High CD36 expression is frequently correlated with aggressive clinicopathological characteristics such as high tumor proliferation, metastasis, vascular invasion, poor differentiation, recurrence, and poor prognosis in patients [17]. Some studies further confirm that CD36 may serve as a potential diagnostic and prognostic biomarker associated with metabolic adaptation and tumor progression in HCC (Table 1).

**Table 1:** Representative studies investigating the role of CD36 in hepatocellular carcinoma. HCC: hepatocellular carcinoma; TNM: tumor-node-metastasis; ATP: adenosine triphosphate; TCGA: The Cancer Genome Atlas; GEO: Gene Expression Omnibus.

References	Study Type	Experimental Model	Main Molecular Focus	Major Findings	Clinical or Translational Significance
Pascual et al. (2017) [8]	Clinical observational study	HCC patient tissues	CD36 expression and prognosis	CD36 expression was significantly elevated in HCC tissues and positively associated with vascular invasion and advanced TNM stage	Suggested CD36 as a potential prognostic biomarker for aggressive HCC
Wang and Li (2019) [10]	Multi-omics investigation	Single-cell transcriptomic	Tumor heterogeneity	CD36-positive tumor populations exhibited immunosuppressive and stemness-associated phenotypes	Revealed potential role in precision oncology
Cheng et al. (2024) [11]	In vitro mechanistic study	HepG2 and Huh7 cells	Fatty acid uptake and proliferation	CD36-mediated fatty acid uptake enhanced lipid accumulation, ATP production, and tumor cell proliferation	Demonstrated metabolic dependency of HCC cells on exogenous lipids
Feng et al. (2023) [12]	Drug resistance analysis	Sorafenib-resistant HCC cells	Therapeutic resistance	Increased CD36 expression enhanced metabolic flexibility and resistance to sorafenib treatment	Suggested CD36 inhibition may improve therapeutic response
Ruan et al. (2022) [13]	Mechanistic investigation	HCC stem-like cells	Cancer stemness	CD36 activation promoted stem cell-like phenotypes and tumor recurrence	Potential target for reducing relapse and metastasis
Silverstein and Febbraio (2009) [15]	Hypoxia-related study	Hypoxic HCC microenvironment	Metabolic adaptation	CD36 expression increased under hypoxic conditions and promoted fatty acid oxidation	Facilitated survival under nutrient stress
Jiang et al. (2021) [26]	Translational study	Human HCC tissues and cell models	Ferroptosis regulation	CD36 altered lipid peroxide accumulation and ferroptosis sensitivity	Identified CD36 as a regulator of oxidative stress adaptation
Yang et al. (2023) [28]	Bioinformatics analysis	TCGA and GEO datasets	Metabolic gene signatures	Elevated CD36 expression was correlated with lipid metabolic pathways and poor overall survival	Supported prognostic and predictive value of CD36
Xu et al. (2021) [30]	Immunometabolism study	Tumor-associated macrophages and T cells	Immune suppression	CD36-mediated lipid accumulation impaired cytotoxic T-cell activity and enhanced M2 macrophage polarization	Suggested role as a metabolic-immune checkpoint

CD36-Mediated Lipid Metabolism Reprogramming in HCC

This review provides evidence that CD36 is a key regulator of fatty acid uptake and intracellular lipid accumulation in HCC cells. Increased CD36 expression enhances cellular adaptation to the hypoxic and nutrient-limited microenvironment by promoting the uptake of exogenous long-chain fatty acids, thereby promoting lipid metabolic plasticity.

Multiple studies have shown that CD36-mediated lipid accumulation promotes membrane biosynthesis, ATP generation, oxidative stress tolerance, and tumor survival under metabolic stress [18]. The role of lipid metabolic reprogramming by CD36 is tumor microenvironment-dependent, with some cancers benefiting and others suffering from ferroptosis. The availability and composition of fatty acids, antioxidant capability, and the tumor microenvironment all influence the cellular metabolic state as a result of CD36-mediated lipid metabolic reprogramming. This context dependence might account for apparently conflicting consequences, highlighting that CD36 cannot be viewed solely as an oncogenic lipid transporter. Instead, its biological effects should be considered in relation to the broader metabolism and immunology of HCC. CD36 interacts with major metabolic pathways, including glycolysis, β-oxidation, lipid synthesis, and mitochondrial metabolism. These metabolic changes contribute to rapid tumor growth, metastasis, and adaptation to therapeutic stress.

Furthermore, CD36-related metabolic reprogramming is closely related to the regulation of reactive oxygen species (ROS) and redox homeostasis. Multiple studies have shown that alterations in lipid metabolism promote adaptation to oxidative stress and enhance tumor cell survival under harsh microenvironmental conditions [18].

Molecular Mechanisms of CD36 in HCC Progression

Included literature indicates that CD36 activates multiple oncogenic signaling pathways involved in HCC progression [19]. Previous studies have shown that fatty acid-dependent CD36 can stimulate signaling pathways such as PI3K/AKT/mTOR, MAPK, STAT3, TGF-β, and Wnt/β-catenin, thereby promoting cell proliferation, migration, invasion, angiogenesis, and epithelial-mesenchymal transition (EMT) (Table 2).

**Table 2:** CD36-Regulated signaling pathways and their biological functions in HCC. EMT: epithelial-mesenchymal transition; TGF-β: transforming growth factor beta; STAT3: signal transducer and activator of transcription 3; SREBP: sterol regulatory element-binding protein; MAPK: mitogen-activated protein kinase; ERK: extracellular signal-regulated kinase; HIF-1α: hypoxia-inducible factor 1 alpha; PI3K: phosphoinositide 3-kinase; AKT: protein kinase B; mTOR: mechanistic target of rapamycin; AMPK: AMP-activated protein kinase; PPAR: peroxisome proliferator-activated receptor; PPARα: peroxisome proliferator-activated receptor alpha; FAO: fatty acid oxidation; ATP: adenosine triphosphate; NF-κB: nuclear factor kappa B; ROS: reactive oxygen species.

References	Signaling Pathway	Upstream Trigger	CD36-Associated Molecular Events	Biological Effects	Clinical Implications
Pascual et al. (2017) [8]	Wnt/β-catenin	Metabolic plasticity and lipid remodeling	Activation of stemness‑related transcriptional programs	EMT induction and cancer stem cell maintenance	Linked to recurrence and poor prognosis
Pascual et al. (2017) [8]	TGF‑β	Tumor microenvironment remodeling	Regulation of fibrosis and EMT‑associated genes	Enhanced invasiveness and metastatic behavior	Associated with aggressive tumor phenotype
Pascual et al. (2017) [8]	STAT3	Chronic inflammation and cytokine signaling	Increased inflammatory and survival signaling	Immune evasion and tumor persistence	Potential target for combined metabolic‑immunotherapy
Cheng et al. (2024) [11]	SREBP‑mediated signaling	Lipid biosynthesis activation	Increased cholesterol and fatty acid synthesis	Membrane biosynthesis and metabolic remodeling	Supports rapid tumor growth
Pascual et al. (2017) [8]; Feng et al. (2023) [12]	MAPK/ERK	Lipid‑mediated signaling activation	Increased phosphorylation of ERK‑related pathways	Enhanced migration, invasion, and proliferation	Associated with metastatic progression
Silverstein and Febbraio (2009) [15]	HIF‑1α pathway	Hypoxia and nutrient deprivation	Increased fatty acid metabolic adaptation	Enhanced tumor survival under hypoxic conditions	Promotes aggressive tumor microenvironment
Silverstein and Febbraio (2009) [15]	PI3K/AKT/mTOR	Fatty acid uptake and nutrient stress	Activation of survival and anabolic signaling pathways	Enhanced proliferation, survival, and metabolic adaptation	Promotes tumor progression and therapeutic resistance
Cheng et al. (2024) [11]; Yang et al. (2023) [28]	AMPK/PPAR signaling	Energy stress and fatty acid oxidation	Regulation of lipid metabolism and mitochondrial adaptation	Metabolic flexibility and oxidative stress tolerance	Facilitates survival under hypoxia
Cheng et al. (2024) [11]; Yang et al. (2023) [28]	PPARα/FAO axis	Fatty acid oxidation induction	Enhanced mitochondrial β‑oxidation	ATP production and energy homeostasis	Supports therapeutic resistance
Xu et al. (2021) [30]	NF‑κB signaling	Inflammatory stress and ROS accumulation	Activation of pro‑inflammatory transcriptional responses	Chronic inflammation and tumor progression	Contributes to immune suppression and resistance

Some studies have also highlighted the role of CD36 in maintaining cancer stem cell characteristics and metastatic colonization [20]. Increased CD36 expression is associated with enhanced tumor recurrence, metastatic ability, and anti-apoptotic capacity. However, some studies have reported context-dependent CD36 function under different metabolic conditions, suggesting that the biological role of CD36 may vary depending on tumor stage, nutrient supply, and microenvironment composition.

CD36, Ferroptosis, and Oxidative Stress Regulation

Increasing evidence suggests that CD36 is an important regulator of fermentation and lipid peroxidation in HCC (Table 3). CD36-mediated polyunsaturated fatty acid uptake promotes the accumulation of lipid peroxides and modulates ferroptosis sensitivity. Multiple studies have shown that CD36 dysregulation affects the balance between pro-survival antioxidant defense and ferroptosis pathways [21].

**Table 3:** Role of CD36 in lipid metabolic reprogramming, ferroptosis, and oxidative stress. HCC: hepatocellular carcinoma; NADPH: nicotinamide adenine dinucleotide phosphate.

References	Biological Process	CD36-Associated Mechanism	Cellular Consequences	Impact on HCC Progression
Pascual et al. (2017) [8]; Wang and Li (2019) [10]; [11] Cheng et al. (2024)	Fatty acid uptake	Increased transport of long-chain fatty acids	Intracellular lipid accumulation	Supports rapid tumor growth
Cheng et al. (2024) [11]	Lipid metabolic plasticity	Crosstalk with glycolysis and β-oxidation	Enhanced metabolic adaptation	Survival under nutrient deprivation
Cheng et al. (2024) [11]	Mitochondrial dysfunction	Altered oxidative phosphorylation	Increased metabolic stress tolerance	Enhanced tumor survival
Tang et al. (2021) [14]	Glutathione metabolism	Crosstalk with antioxidant pathways	Prevention of excessive lipid oxidation	Maintenance of tumor viability
Silverstein and Febbraio (2009) [15]	Hypoxia‑associated metabolism	Adaptation to oxygen deprivation	Increased metabolic flexibility	Aggressive tumor phenotype
Tang et al. (2021) [14]; Jiang et al. (2021) [26]	Iron-dependent ferroptosis	Interaction with ferroptosis‑related signaling	Modulation of ferroptotic cell death	Potential therapeutic vulnerability
Tang et al. (2021) [14]; Jiang et al. (2021) [26]	Lipid peroxidation	Accumulation of polyunsaturated fatty acids	Enhanced ferroptotic sensitivity or resistance	Context‑dependent tumor regulation
Tang et al. (2021) [14]; Jiang et al. (2021) [26]	Reactive oxygen species regulation	Altered mitochondrial metabolism and lipid oxidation	Oxidative stress adaptation	Increased resistance to hostile microenvironment
Jiang et al. (2021) [26]; Yang et al. (2023) [28]	Antioxidant defense pathways	Regulation of redox homeostasis	Protection from oxidative damage	Tumor persistence and therapy resistance
Jiang et al. (2021) [26]; Yang et al. (2023) [28]	NADPH homeostasis	Regulation of redox balance	Reduced oxidative injury	Increased resistance to therapy

This review further confirms that CD36 participates in oxidative stress adaptation by regulating reactive oxygen species (ROS) production, mitochondrial function, and antioxidant signaling pathways. However, the exact role of CD36 in inducing or inhibiting ferroptosis remains unclear, with responses depending on the cellular metabolic state and microenvironment composition.

CD36 and Tumor Immunometabolism

Analytical studies have shown that CD36 significantly promotes the remodeling of the tumor immune microenvironment in HCC (Table 4). Lipid over-uptake via CD36 results in intracellular lipid accumulation in immune cells, resulting in promoted activity of cytotoxic T cells, T-cell exhaustion, and macrophage polarization.

**Table 4:** CD36-Mediated immunometabolic remodeling in the HCC tumor microenvironment. HCC: hepatocellular carcinoma.

References	Immune Component	CD36-Associated Alteration	Functional Outcome	Clinical Relevance
Pascual et al. (2017) [8]	Tumor stromal interactions	Altered metabolic communication	Remodeling of tumor microenvironment	Increased metastatic potential
Zhang et al. (2026) [9]	Dendritic cells	Impaired antigen presentation	Reduced T-cell activation	Weak antitumor immunity
Zhang et al. (2026) [9]	Chronic inflammatory signaling	Persistent cytokine production	Immune dysregulation and tumor persistence	Poor patient prognosis
Zhang et al. (2026) [9]	Natural killer cells	Lipid-induced functional suppression	Reduced cytotoxic capacity	Impaired tumor clearance
Cheng et al. (2024) [11]	Tumor-associated macrophages	Promotion of M2 polarization	Immunosuppressive microenvironment	Enhanced tumor progression
Xu et al. (2021) [30]	Cytotoxic CD8+ T cells	Lipid accumulation and metabolic exhaustion	Reduced antitumor activity	Impaired immunotherapy response
Xu et al. (2021) [30]	Immune checkpoint pathways	Crosstalk with metabolic signaling	Reduced sensitivity to immune checkpoint blockade	Therapeutic resistance
Xu et al. (2021) [30]	Inflammatory cytokine signaling	Enhanced inflammatory pathway activation	Chronic tumor-promoting inflammation	Disease progression
Xu et al. (2021) [30]	Myeloid-derived suppressor cells	Metabolic activation and accumulation	Enhanced immune suppression	Poor therapeutic response
Xu et al. (2021) [30]	Regulatory T cells	Altered metabolic adaptation	Suppressed immune surveillance	Increased immune evasion

Multiple studies have confirmed that CD36 is a potential metabolic immune checkpoint involved in immune escape and attenuated responses to immunotherapy [22]. CD36-mediated metabolic competition between tumor cells and immune cells impacts inflammatory signaling, immune surveillance, and antitumor immune responses. The findings imply that targeting immunometabolic pathways involving CD36 could enhance the effectiveness of immune checkpoint blockade (ICB) in HCC.

CD36-Mediated Drug Resistance

Literature reviews consistently indicate a close relationship between CD36-related metabolic adaptation and treatment resistance in HCC (Table 1). Upregulation of CD36 contributes to increased resistance to chemotherapy, targeted therapies, and immunotherapy through the development of metabolic flexibility, resistance to oxidative stress, upregulation of survival signaling, and tumor stemness.

Previous studies have shown that activation of compensatory metabolic pathways and stress response signaling networks is associated with CD36-mediated resistance to sorafenib and lenvatinib [23]. Experimental studies also indicate that blocking CD36 may sensitize tumors to treatment, enhance immune repair potential, and enhance antitumor immune responses [24].

Emerging Therapeutic Strategies Targeting CD36

Recent preclinical studies have shown that pharmacological inhibition or gene silencing of CD36 can reduce fatty acid uptake, inhibit tumor growth, decrease metastatic potential, and improve sensitivity to anticancer therapies (Table 5) [25]. Experimental models of HCC have shown combination therapy with CD36 inhibitors, ferroptosis inducers, immune checkpoint inhibitors, and nanotechnology-based drug delivery systems to be effective against tumor cells.

**Table 5:** Therapeutic strategies targeting CD36 in hepatocellular carcinoma.

References	Therapeutic Strategy	Representative Approaches	Mechanism of Action	Antitumor Effects	Current Research Status
Wang and Li (2019) [10]; Feng et al. (2023) [12]	Monoclonal antibodies	Anti‑CD36 antibodies	Blockade of CD36 signaling and ligand interaction	Suppressed tumor growth and invasion	Experimental
Wang and Li (2019) [10]	Small‑molecule inhibitors	Sulfo‑N‑succinimidyl oleate (SSO), T0901317	Inhibition of fatty acid uptake	Reduced proliferation and metastasis	Preclinical
Feng et al. (2023) [12]	CRISPR/Cas‑based approaches	CD36 gene editing	Permanent suppression of CD36 activity	Potential long‑term therapeutic benefit	Early‑stage research
Feng et al. (2023) [12]	Monoclonal antibodies	Anti‑CD36 antibodies	Blockade of CD36 signaling and ligand interaction	Suppressed tumor growth and invasion	Experimental
Ruan et al. (2022) [13]	Gene‑silencing strategies	siRNA/shRNA‑mediated CD36 suppression	Downregulation of oncogenic signaling	Inhibition of tumor progression	Experimental
Ruan et al. (2022) [13]	Nanoparticle‑mediated delivery	Lipid‑based nanoparticles	Targeted drug delivery to HCC tissues	Improved therapeutic efficiency	Emerging
Ruan et al. (2022) [13]	Precision nanomedicine	CD36‑targeted multifunctional nanoparticles	Selective tumor‑targeted therapy	Reduced off‑target toxicity	Emerging precision oncology approach
Ruan et al. (2022) [13]	Small‑molecule inhibitors	Sulfo‑N‑succinimidyl oleate (SSO), T0901317	Inhibition of fatty acid uptake	Reduced proliferation and metastasis	Preclinical
Yang et al. (2023) [28]	Targeted metabolic therapy	Combined inhibition of lipid metabolism pathways	Suppression of metabolic plasticity	Reduced resistance to therapy	Translational research stage
Huang et al. (2024) [32]	Sorafenib combination therapy	CD36 inhibition plus sorafenib	Restoration of drug sensitivity	Reduced therapeutic resistance	Preclinical

In addition, recent progress in lipidomics, metabolomics, single-cell sequencing, and spatial transcriptomics has substantially enriched the understanding of the metabolic and signaling heterogeneity of HCC and CD36 signaling networks (Table 6). These emerging technologies hold promise for advancing biomarker development, patient stratification, and the formulation of precision oncology treatment strategies targeting metabolic dysfunction in HCC.

**Table 6:** Emerging multi-omics technologies and precision medicine approaches in CD36-related HCC research. RNA: ribonucleic acid; HCC: hepatocellular carcinoma.

References	Technology	Research Application	Major Findings	Translational Importance
Wang and Li (2019) [10]	Single‑cell RNA sequencing	Cellular heterogeneity analysis	Identification of CD36‑positive tumor and immune subpopulations	Precision patient stratification
Cheng et al. (2024) [11]	Lipidomics	Lipid species characterization	Identification of altered lipid metabolic signatures	Discovery of metabolic biomarkers
Feng et al. (2023) [12]	Proteomics	Protein interaction analysis	Identification of CD36‑associated regulatory proteins	Mechanistic pathway discovery
Yang et al. (2023) [28]	Metabolomics	Metabolic pathway profiling	Detection of altered fatty acid oxidation and redox metabolism	Therapeutic target identification
Ally et al. (2017) [33]	Artificial intelligence‑assisted analysis	Predictive biomarker modeling	Identification of prognostic metabolic signatures	Precision oncology development
Ally et al. (2017) [33]	Bulk RNA sequencing	Transcriptomic profiling	Identification of CD36‑associated gene signatures	Prognostic biomarker development
Ally et al. (2017) [33]	Digital pathology	Histopathological image analysis	Correlation of CD36 expression with tumor morphology	Improved diagnostic evaluation
Ally et al. (2017) [33]	Machine learning approaches	Predictive therapeutic modeling	Identification of metabolic response signatures	Personalized treatment optimization
Ally et al. (2017) [33]	Multi‑omics integration	Cross‑platform biological analysis	Comprehensive mapping of metabolic signaling networks	Improved systems‑level understanding
Ally et al. (2017) [33]	Spatial transcriptomic	Tumor microenvironment mapping	Localization of CD36‑associated signaling networks	Understanding tumor spatial heterogeneity

Discussion

This review highlights the increasingly important role of CD36 in metabolic reprogramming and tumor progression in HCC. A growing body of evidence suggests that CD36 functions not only as a fatty acid transporter but also as a multifunctional regulator involved in lipid metabolism, oxidative stress adaptation, ferroptosis regulation, immune remodeling, and treatment resistance. The synergistic effects between these biological processes indicate that CD36 is associated with the invasion, metastatic potential, and poor clinical prognosis of HCC [26].

One of the key findings is the strong association between CD36 expression and metabolic plasticity in HCC cells. Tumor cells undergo extensive metabolic adaptations to survive under hypoxic and nutrient-deficient conditions, and CD36 appears to promote these processes by increasing the uptake and utilization of exogenous fatty acids. Tumor cells can adapt to microenvironmental stresses and continue to proliferate due to these CD36-mediated metabolic adjustments, supporting the aforementioned molecular results. Multiple studies have further confirmed that CD36-mediated metabolic adaptation enhances tumor survival under oxidative and therapeutic stress conditions [15]. The interaction between CD36 and oncogenic signaling pathways is another important mechanism that promotes HCC progression. A growing body of research suggests that CD36-mediated lipid metabolism remodeling activates a number of oncogenic pathways, including the PI3K/AKT/mTOR, MAPK/ERK, STAT3, Wnt/β-catenin, and TGF-β signaling pathways. These pathways collectively regulate cell proliferation, epithelial-mesenchymal transition (EMT), angiogenesis, stem cell induction, stem cell maintenance, and metastatic colonization. Importantly, lipid metabolic signaling pathways and oncogenic pathways appear to be cross-directional, suggesting that metabolic reprogramming is tightly integrated with the tumor signaling network during HCC progression [8].

Recent studies have also revealed the important role of CD36 in the regulation of fermentation and adaptation to oxidative stress [27]. Ferroptosis is characterized by iron-dependent lipid peroxidation, and CD36 binding to polyunsaturated fatty acids may have a crucial influence on cellular sensitivity to ferroptosis. Some studies have shown that increased CD36 expression enhances the accumulation of lipid peroxides and induces oxidative damage, while others have shown that CD36 promotes antioxidant defense mechanisms and oxidative resistance by maintaining mitochondrial redox balance and adaptation. These seemingly contradictory findings may be related to metabolic heterogeneity, tumor cell differences, and microenvironment variation in HCC tissues [28]. This dual function creates a fundamental paradox for CD36-targeted therapy: blocking CD36 may make tumors more susceptible to ferroptosis by limiting the uptake of polyunsaturated fatty acids and the buildup of lipid peroxides, but it may also remove a key cause of oxidative stress that could be used therapeutically. In contrast, boosting CD36 activity in some conditions might accelerate ferroptosis, but it may also activate antioxidant defense mechanisms that protect the tumor cells from oxidative damage. This conflict is further complicated by the fact that CD36-mediated lipid accumulation can have both pro-survival and pro-ferroptosis effects depending on the cellular redox state, antioxidant capability, and tumor microenvironment composition [10]. Therefore, the therapeutic potential of targeting CD36 may not be considered an ordinary reversible procedure; rather, it requires careful assessment of the specific combination of therapy approach used, the tumor metabolic setting, and baseline ferroptosis susceptibility.

Another significant finding is the growing recognition of the metabolic and immunomodulatory role of CD36 in the tumor microenvironment. CD36-mediated lipid over-uptake promotes lipid accumulation in immune cells, particularly cytotoxic CD8+ T cells and tumor-associated macrophages, thereby impairing antitumor immune responses [29]. CD36-related immunometabolic remodeling leads to T-cell exhaustion, macrophage M2 polarization, chronic inflammatory signaling, and immune escape. Therefore, several recent studies have suggested that CD36 may be a potential metabolic immune checkpoint involved in reducing the efficacy of immunotherapy and immunotherapy-blocking therapies [30].

Treatment resistance remains one of the major clinical challenges in the treatment of HCC, and mounting evidence suggests that CD36-related metabolic adaptation is closely associated with resistance to chemotherapy, targeted therapy, and immunotherapy. Increased CD36 expression promotes metabolic flexibility and oxidative stress tolerance and activates survival signaling pathways under treatment-induced stress [31]. In particular, CD36 is closely associated with resistance to sorafenib and lenvatinib, with mechanisms involving compensatory metabolic reprogramming and stress adaptation. Experiments have shown that inhibiting CD36 has good antitumor effects, including inhibiting cell proliferation, restoring therapeutic sensitivity, inducing ferroptosis, and enhancing antitumor immune activity (Table 5) [32].

The rapid development of multi-omics technologies has greatly improved our understanding of the CD36-related metabolic network in HCC. Advances in single-cell RNA sequencing, spatial transcriptomics, lipidomics, metabolomics, and AI-assisted analysis have enabled us to more comprehensively characterize tumor heterogeneity and metabolic adaptation. These technologies have potential for the discovery of CD36-associated biomarkers for prognostic prediction, treatment response evaluation, and patient stratification. Moreover, multi-omics integration can offer new opportunities for precision oncology approaches for metabolic dysfunction in HCC [33] (Table 6).

Despite significant progress, some gaps and uncertainties remain in the existing literature. Most of the current studies are performed using experimental models, and large-scale clinical validation studies are limited. Moreover, the context-dependent function of CD36 in defense regulation, immune remodeling, and metabolic adaptation needs to be further elucidated. Current challenges remain in translating lipid metabolism signaling pathways within the tumor microenvironment into clinical applications. Thus, there is a need for further studies to validate the mechanisms, develop biomarkers, and optimize combination therapy strategies targeting CD36-related metabolic pathways in HCC [34].

In summary, current evidence supports CD36 as a key mediator in HCC, linking lipid metabolism, oxidative stress, immune remodeling, and treatment resistance. A deeper understanding of the mechanisms of CD36-related metabolic signaling may provide new opportunities for developing metabolism-based precision therapies and improving clinical management strategies for patients with HCC [11].

## Conclusions

This review demonstrates that CD36 plays a crucial, multifunctional regulatory role in HCC, linking lipid metabolism regulation, immune evasion, ferroptosis regulation, and treatment resistance. Extensive preclinical evidence suggests that CD36 overexpression is closely linked with tumor invasion, increased metastasis, and poor prognosis. Notably, CD36-dependent fatty acid uptake activates oncogenic pathways and remodels the tumor immune microenvironment via T-cell dysfunction and macrophage polarization, while its role in ferroptosis regulation remains unclear. These findings suggest that CD36 is a prominent metabolic-immune checkpoint and a potential biomarker for patient stratification. However, the clinical translation of CD36-targeting strategies remains limited by its tissue-specific expression, lack of large-scale validation, and concerns about systemic toxicity. This review highlights the importance of CD36 as a therapeutic target and the need for more high-quality research to establish the safety and efficacy of intervention strategies. Future perspectives suggest that while CD36 inhibition offers an alternative therapeutic framework, treatment should be individualized based on tumor metabolic heterogeneity and the microenvironment, and multi-omics technologies should be integrated for biomarker-guided patient selection and optimization of combination therapy regimens, especially in high-risk HCC populations.
